# Supplementary material for: Machine learning algorithms for predicting coronary artery disease: efforts toward an open source solution
Source: Future Sci OA. 2021 Mar 29;7(6):FSO698. doi: 10.2144/fsoa-2020-0206 (PMC8147740; doi:10.2144/fsoa-2020-0206)
Supplement: Supplementary file 1 [file fsoa-07-698-s1.pdf]

Supplemental Material

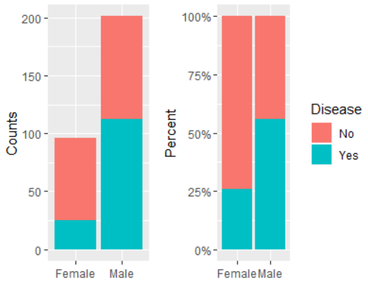

S1: Patients with and without heart disease.

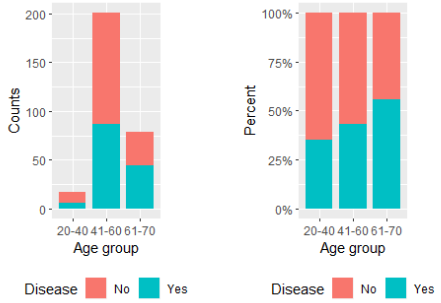

S2: Age of patients with and without heart disease.

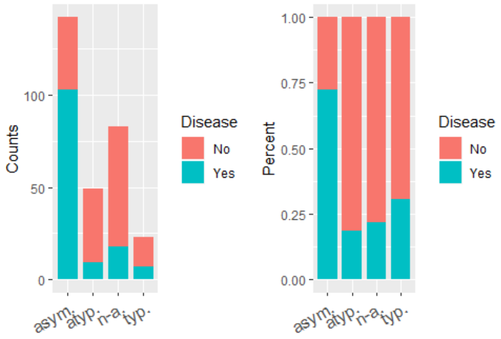

S3: Type of chest pain in patients with and without heart disease. Abbreviations: typ., typical angina; atyp., atypical angina; n-a., non-anginal pain; asym., asymptotic

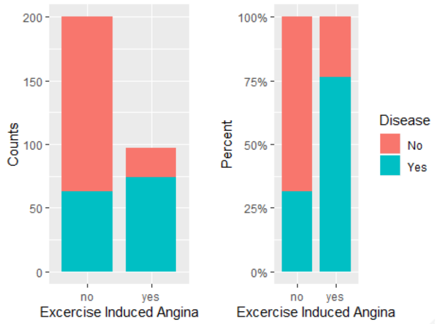

S4: Exercise induced angina (EIA) in patients with and without heart disease.

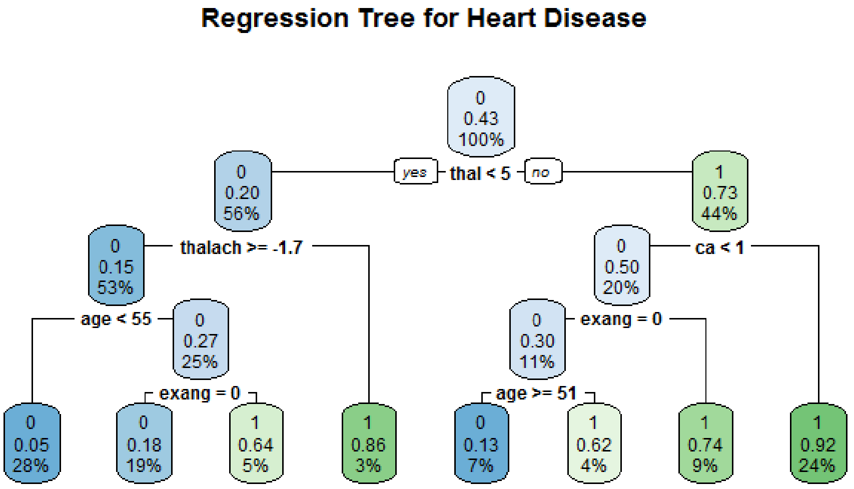

S5: Regression Tree Model

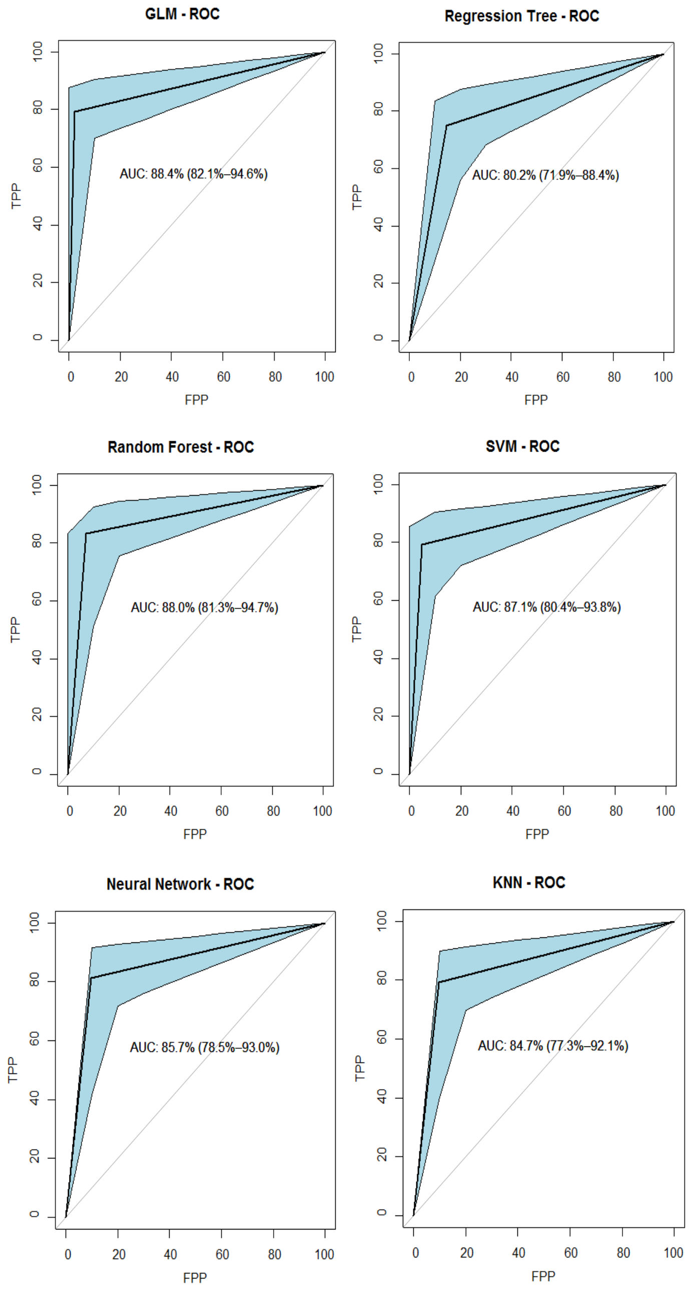

S6: Composite AUC-ROC curves
