# Supplementary material for: Machine learning algorithms for predicting coronary artery disease: efforts toward an open source solution
Source: Future Sci OA. 2021 Mar 29;7(6):FSO698. doi: 10.2144/fsoa-2020-0206 (PMC8147740; doi:10.2144/fsoa-2020-0206)
Supplement: Supplementary file 2 [file fsoa-07-698-s2.docx]

**Related Works**:

Abdar, M., Kalhori, S. R. N., Sutikno, T., Subroto, I. M. I., & Arji, G. (2015). Comparing Performance of Data Mining Algorithms in Prediction Heart Diseases. *International Journal of Electrical & Computer Engineering (2088-8708)*, *5*(6).

Abdar, Moloud. "Using Decision Trees in Data Mining for Predicting Factors Influencing of Heart Disease." *Carpathian Journal of Electronic & Computer Engineering* 8, no. 2 (2015).

Abdar, M., Acharya, U. R., Sarrafzadegan, N., & Makarenkov, V. (2019). NE-nu-SVC: A new nested ensemble clinical decision support system for effective diagnosis of coronary artery disease. *IEEE Access*, *7*, 167605-167620.

Abdar, M., Książek, W., Acharya, U. R., Tan, R. S., Makarenkov, V., & Pławiak, P. (2019). A new machine learning technique for an accurate diagnosis of coronary artery disease. *Computer methods and programs in biomedicine*, *179*, 104992.

Ahmed, H., Younis, E. M. G., Hendawi, A., & Ali, A. A. (2019). *Heart disease identification from patients’ social posts, machine learning solution on Spark. Future Generation Computer Systems.* doi:10.1016/j.future.2019.09.056

Alkeshuosh, A. H., Moghadam, M. Z., Mansoori, I. A., & Abdar, M. (2017). *Using PSO Algorithm for Producing Best Rules in Diagnosis of Heart Disease. 2017 International Conference on Computer and Applications (ICCA).* doi:10.1109/comapp.2017.8079784

Atallah, R., & Al-Mousa, A. (2019). *Heart Disease Detection Using Machine Learning Majority Voting Ensemble Method. 2019 2nd International Conference on New Trends in Computing Sciences (ICTCS).* doi:10.1109/ictcs.2019.8923053

Aouabed Z, Abdar M, Tahiri N. A Novel Effective Ensemble Model for Early Detection of Coronary Artery Disease A Novel Effective Ensemble Model for Early Detection of Coronary Artery Disease. 2020;(January). *International Conference Europe Middle East & North Africa Information Systems and Technologies to Support Learning*. Springer, Cham, 2019. doi:10.1007/978-3-030-36778-7

Garate-Escamilla, Anna Karen, Amir Hajjam EL Hassani, and Emmanuel Andres. "Classification models for heart disease prediction using feature selection and PCA." *Informatics in Medicine Unlocked* (2020): 100330.

Garg S.B., Rani P., Garg J. (2021) Performance Analysis of Classification Methods in the Diagnosis of Heart Disease. In: Marriwala N., Tripathi C.C., Kumar D., Jain S. (eds) Mobile Radio Communications and 5G Networks. Lecture Notes in Networks and Systems, vol 140. Springer, Singapore. https://doi.org/10.1007/978-981-15-7130-5_58

Gupta, A., Kumar, L., Jain, R., & Nagrath, P. (2020). Heart Disease Prediction Using Classification (Naive Bayes). In *Proceedings of First International Conference on Computing, Communications, and Cyber-Security (IC4S 2019)* (pp. 561-573). Springer, Singapore.

Mienye, Ibomoiye Domor, Yanxia Sun, and Zenghui Wang. "An improved ensemble learning approach for the prediction of heart disease risk." *Informatics in Medicine Unlocked* 20 (2020): 100402.

Mujeeb, S. M., Praveen Sam, R., & Madhavi, K. (2020). *Adaptive hybrid optimization enabled stack autoencoder-based MapReduce framework for big data classification. 2020 International Conference on Emerging Trends in Information Technology and Engineering (ic-ETITE).* doi:10.1109/ic-etite47903.2020.6366147

Ramalingam, V. V., Dandapath, A., & Raja, M. K. (2018). Heart disease prediction using machine learning techniques: a survey. *International Journal of Engineering & Technology*, *7*(2.8), 684-687.

Zomorodi‐moghadam, M., Abdar, M., Davarzani, Z., Zhou, X., Pławiak, P., & Acharya, U. R. (2019). *Hybrid particle swarm optimization for rule discovery in the diagnosis of coronary artery disease. Expert Systems.* doi:10.1111/exsy.12485
